# The influence of ventilation modes on oil mist particles diffusion in a machining workshop

**DOI:** 10.1016/j.heliyon.2024.e26963

**Published:** 2024-02-26

**Authors:** Yilin Li, Shiji Zong, Huaiwang Jing, Nan Gao, Heping Ye, Jianbo Chen

**Affiliations:** School of Environment and Architecture, University of Shanghai for Science and Technology, Shanghai, China

**Keywords:** Ventilation mode, Oil mist, Particles concentration, Machining workshop

## Abstract

Mechanical processing and operations are widely involved in modern industry. Large amount of oil mist is tended to be produced and will diffuse in the processing workshop when metalworking fluids are applied on the high temperature workpiece. The ventilation modes and air distributions can influence the air pollutants dilution in machining workshops. Therefore, this paper presents both experimental investigation and simulation study on the oil mist particles diffusion under different ventilation modes. The results identified PM2.5 as the primary component among different oil mist particles generated during a typical machining process. The distribution of oil mist particles in a full-scale machining workshop laboratory was investigated under two ventilation modes: high-sidewall nozzle air supply and low-sidewall air supply. Results revealed obvious influences of both air supply modes on the distribution of oil mist particles. Under the high-sidewall-nozzle air supply mode, the airflow and the oil mist distribution in the workshop was relatively uniform; while the low-sidewall-vent air supply mode led to an uneven distribution of oil mist particles, and the maximum oil mist concentration appeared at the height of 3 m. Under both modes, the attempts to increase the airflow rate are not always successful. Compared with low-sidewall-vent air supply mode, the high-sidewall-nozzle air supply mode presents better performance in achieving lower overall particle concentration level. Overall, the results of this study give useful reference to improve the air quality of industrial plant by properly designing the ventilation mode of machining workshop.

## Highlights

• PM2.5 dominates oil mist particle components during typical machining process.
• HSN air supply mode provides uniform oil mist distribution in machining workshop.
• LSV air supply mode leads to unevenly distributed oil mist in machining workshop.
• Lower overall particle concentration level can be achieved by HSN air supply mode.

## Nomenclature

ρ: the density of oil mist particles
j: 1, 2, 3, representing x, y, z three components respectively
t: the time of motion
S: quality original term
v: the velocity of the oil mist particles (m/s)
$F_{j}$: the component of the external body force in the j direction
$g_{j}$: the component of gravity acceleration in j direction
p: static pressure
$\tau_{ij}$: the stress tensor at the fluid element
η: ventilation efficiency (%)
MWFs: Metalworking fluids
VOC: Volatile organic compounds
OSHA: Occupational Safety and Health Administration
MQL: Minimum quantity lubrication
SEV: Supply and exhaust ventilation
HSN: High-sidewall-nozzle
LSV: Low-sidewall-vent

## 1 Introduction

Mechanical processing and operations play a significant role in modern industry, offering advantages such as reducing human errors, improving production efficiency and product quality, and saving costs [1,2]. However, machining processing often leads to the generation of excessive heat between the tool and the workpiece. To address this issue and enhance tool life while minimizing thermal deformation of the workpiece, metalworking fluids (MWFS) are commonly used for lubrication and cooling [3]. Nevertheless, the application of MWFs on high-temperature workpieces tends to result in the production of a large amount of oil mist, which then diffuses within the processing workshop [4]. Previous studies have identified two main sources for the generation of oil mist: some oil mist particles are initially formed by the high-pressured ejection of MWFS from the nozzle [5], while the others are generated during the mixing and condensing process of the high-temperature oil vapor with the surrounding cool air [6,7].

Although it is known that the oil mist mainly consists of liquid particles and volatile organic compounds (VOC), many researchers put great effort in investigating the composition of oil mist in actual workshops [8,9]. Results showed that the fine and ultra-fine particles account for a large portion of the oil mist. Dunja S. et al. [[10], [10]] studied the impact of three different industrial MWFs on the particle size of aerosol at oil-in-water emulsion concentrations of 1%, 6% and 10%. They observed that increasing the oil concentration in the emulsion can result in larger particle size of aerosol, while the viscosity of the emulsion had little impact on the particle size.

According to the Occupational Safety and Health Administration (OSHA), the limit of exposure to the airborne mineral oil mist is 0.5 mg/m^3^ over an average 8-h period [11]. Unfortunately, the oil mist levels in many factories have been found to exceed the required value. Due to long-term exposure to the liquid particles and VOC components of the oil mist, occupants in machining workshops are more likely to suffer health hazards than those who work in normal environment [12]. It has been revealed that oil mist particles generated during machining processing often cause symptoms or disease such as asthma, cough, and rhinitis [13,14]. Long-term exposure to oil mist can lead to increased risk of laryngeal cancer, pancreatic cancer, and rectal cancer [15,16]. Therefore, it is essential to remove oil mist in the machining workshop to an acceptable level for achieving a healthier and high-efficiency working environment.

Currently, there are mainly three ways to reduce the oil mist particles levels: source elimination, local exhaust or local purification, and ventilation dilution. In terms of source elimination, reasonable improvement of cutting fluids is most widely adopted and can effectively reduce the generation of oil mist particles and alleviate indoor pollution problems [17]. Zareh-Desari et al. [18] developed and tested a novel environmentally friendly lubricant, and the results showed that nano-lubricants have obvious advantages over conventional liquid lubricants. Meyer et al. [19] concluded that the change of additive combination had a great influence on the thermal load in the contact zone and the performance of the grinding process. In addition, some enterprises and scholars utilized the minimum quantity lubrication (MQL) technique to control the oil mist particles concentration from the source [20,21]. However, MQL technique is not able to remove the massive heat generated during the machining process [22]. Apart from improved cutting fluids, local exhaust and oil mist purification devices can also be used to remove the oil mist concentration such as mechanical exhaust pumps, oil-mist purification units, and protective covers [23,24]. For instance, Chia et al. [25] investigated the effectiveness of local exhaust and improved operation as intervention measures in reducing the airborne bacterial concentration and aerosol particle size distribution of a large precision machinery factory. Huang et al. [26] studied the ventilation performance of a local exhaust hood in an industrial plant, and found that the pollutant removal efficiency is greatly influenced by the airflow boundaries, the flow rate of exhaust hood, and the intensity of pollution sources. Therefore, optimized exhaust hoods such as an annular exhaust hood [27], and an air curtain exhaust hood [28] were developed to achieve improved local ventilation.

Despite the above methods have proved to be effective in reducing the oil mist concentration, source elimination technologies may not be always possible, and local exhaust is difficult in a non-enclosure or conventional milling machinery environment [29]. Thus, it is necessary to investigate the common ventilation modes and air distributions for their potentials of air pollutants dilution in machining workshops [30]. Wei et al. [31] proposed an improved displacement ventilation system to create a clean breathing area for some industrial workshops. Murga et al. [32]raised a hybrid emergency ventilation system using localized push-pull ventilation to improve the installed displacement ventilation system of a representative workshop. Cao et al. [33] developed a new ventilation system (multi-vortex ventilation) with large aspect ratio in industrial workshops with good effect of pollutant removal. Sokolovic et al. [34] studied the ventilation effects of three industrial MWFs aerosols at wind speeds of 1, 3, 6 and 8 m/s. Wang et al. [35] conducted numerical simulations of pollutant diffusion between two adjacent industrial workshops, but they didn't conduct on-site measurements of the diffusion emissions of pollutants, and there was no on-site experimental data to verify the CFD results. Under such circumstances, it is meaningless to study the performance of the ventilation system in the machine shop. Wang [36] measured the environmental quality of different machining workshops on the spot and discussed the emission rate characteristics of oil mist particles in the workshops, providing a reference for future experimental research and providing design parameters for further research on oil mist control in large-space industrial buildings. Yao [37] used measured boundary conditions to numerically simulate the indoor flow field and particle field to study particle transport and the performance of various ventilation strategies in the processing workshop. Above all, existing research on oil mist particle diffusion in machine shops is mostly based on models and is conducted in natural ventilation or local ventilation modes. It lacks comprehensive consideration of the actual processing environment, resulting in a lack of experimental verification and reducing the credibility of the simulation results. Moreover, modern machining workshops are usually highly-sealed and airtight due to the utilization of heating, ventilation, and air-conditioning systems, which adds difficulty in removing oil mist particles. There is relatively little research information of the effectiveness of general ventilation and its various ventilation modes on the oil mist particles diffusion in a machining workshop, and especially the spatial distribution of oil mist under different ventilation schemes.

To this end, the aim of the study is to investigate the diffusion of oil mist particles under mixed ventilation (HSN) and displacement ventilation (LSV) modes in a machining workshop, as two of the most commonly used ventilation modes in machining workshops. Experimental study has been conducted in order to obtain the spatial distribution of different oil mist particles in the workshop. Numerical simulations using ANSYS FLUENT software were also performed to reveal detailed information of the oil mist movement and dispersion. This paper can provide useful suggestions to improve the air quality of industrial plant by properly designing the ventilation mode of machining workshop.

## 2 Experimental set-up

The experiments in this study were divided into two phases. The first-phase experiments were conducted in a real machining workshop in a factory to obtain essential data of components and characteristics of oil mist particles which were generated during a typical machining process. Based on the obtained information of oil mist particles, the second-phase experiments were carried out in a full-scale large space machining workshop for investigating the effect of ventilation modes on the diffusion of oil mist particles.

### 2.1 First-phase case study experiment

**Fig. 1:**
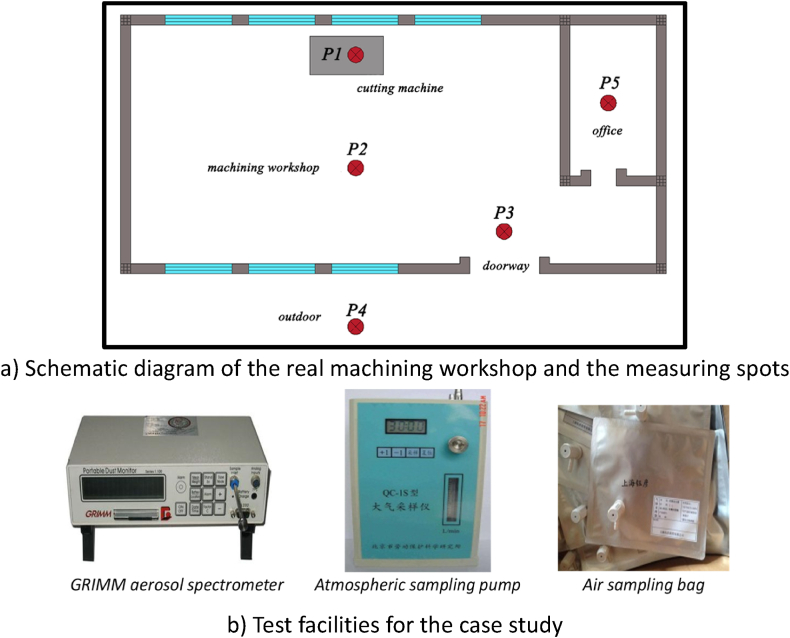
Schematic diagram of the real machining workshop and test facilities.

The first-phase experiments were conducted in an actual machining workshop as a case study (Fig. 1) to obtain the real composition of the oil mist particles generated by machining process. As shown as in Table 1, the machining process was divided into three stages based on the proportion of cutting length and actions of adding chemical fluids, which takes 20 min, and a cylindrical aluminum block (length of 30 cm, radius of 8 cm) was selected as the workpiece. In the P1-1, lubricant was added. The high temperature generated by the friction between the workpiece and the tool makes the upper part of the cutting machine filled with a large amount of mist under the high-speed rotation of the cutting tool. At this time, the concentrations of oil mist particles were measured. In the P1-2, emulsion was added to the lubricant to cool the metal, and then the concentrations of oil mist particles were measured again. During different stages of the machining process and at various positions in the workshop, the concentrations of PM1, PM2.5 and PM10 of oil mist particles were measured. When measuring the concentration of oil mist particles at five locations in the workshop, the time interval between the two measurements was 10 min. The air samples at different measuring spots (P1–P5 in Fig. 1(a)) were collected with sampling bag, and then analyzed by using aerosol spectrometer.

**Table 1:** Sensor location and operation mode.

Measuring spot	Position	Stage of machining process	Duration(min)
P1-1	Above the cutting machine	1) 1/3 cutting length completed; 2) lubricant added	20
P1-2	Above the cutting machine	1) 2/3 length cutting completed; 2) lubricant and emulsion added	
P1-3	Above the cutting machine	Cutting completed	
P2	In the center of the workshop	Cutting completed	10
P3	Near the doorway	Cutting completed	10
P4	Outdoor	Cutting completed	10
P5	In the office	Cutting completed	10

The atmospheric sampling pump and air sampling bags were utilized to collect air samples. The GRIMM aerosol spectrometer can measure the concentration of PM1, PM2.5 and PM10, and other particulate matter in 16 different particle size ranges, and therefore was used to analyze the concentration of the oil mist particles of the air samples. The aerosol spectrometer was set up to collect data every 6 s for 1 min. Therefore, each measuring point has 10 groups of data. The average value of 10 groups of data was calculated to represent the oil mist particle concentration value of the test point. The test facilities and their technical specifications are demonstrated in Fig. 1(b) and Table 2. The obtained components proportions of particulate matter in the oil mist were then used as input parameters of oil mist generator in the second-phase full-scale laboratory experiments.

**Table 2:** Technical specifications of the test facilities.

Device-name	Type	Range	Accuracy	Service conditions
GRIMM	Model-108	0.0001–100 mg/m^3^	±2%	+4∼+45 °C
Aerosol spectrometer				
Atmospheric sampling pump	Type-QC3	0.2–3.0 L/min	±5%	0∼+40 °C
Air sampling bag	Type-2L	0–2 L	±0.05 L	−30∼+45 °C

### 2.2 Second-phase full-scale laboratory experiments

**Fig. 2:**
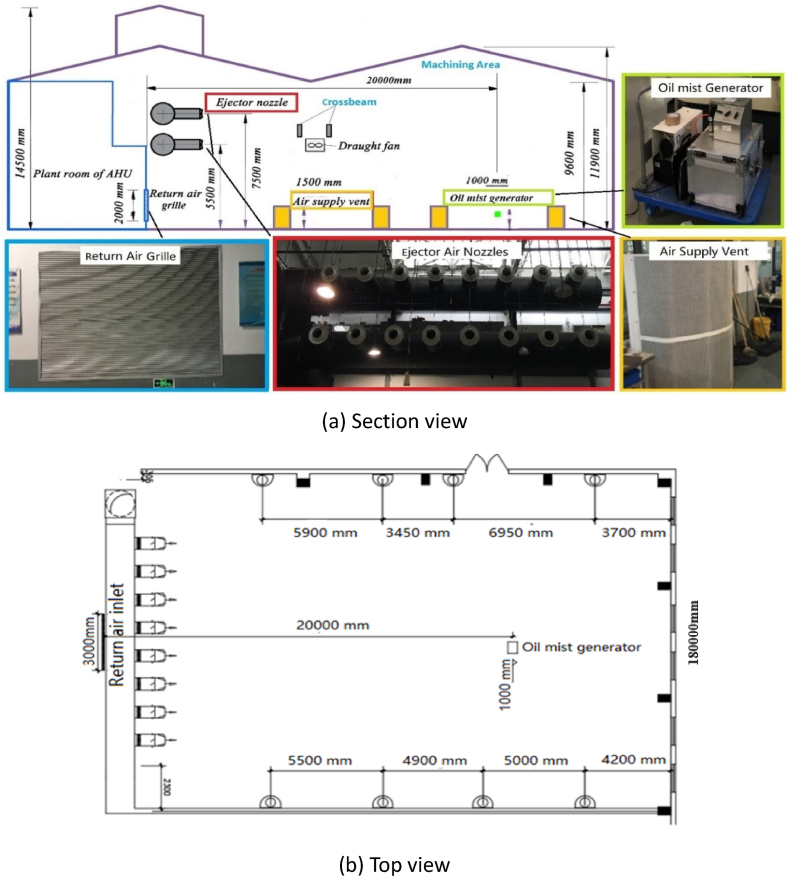
Schematic diagram of the full-scale machining workshop laboratory.

The full-scale machining workshop is located in a large space laboratory in University of Shanghai for Science and Technology, Shanghai, China. The building has a double-sloped roof structure with an area of 648 m^2^ and the total height of the building is 14.5 m. The schematic diagram of the workshop is presented in Fig. 2. There are sixteen air supply pipes which are divided evenly into two rows which are located horizontally near the roof at the height of 5.5 m and 7.5 m respectively. Each air supply pipe is assembled with an air ejector nozzle with a diameter of 0.373 m, and supports the high-sidewall-nozzle (HSN) air supply mode. In addition, there are other eight air pipes arranged vertically in the workshop and divided in halves on the south and north walls respectively. Each wall has four air pipes installed with semi-cylindrical air vents with the height of 1.5 m and radius of 1 m which support the low-sidewall-vent (LSV) air supply mode. The air handling unit (AHU) plant room is located on the eastern part of the workshop and the return air grille is installed on the east wall of the workshop. An oil mist generator was used to simulate the generation process of oil mist particles in a real machining workshop. As shown in Fig. 2(b), the oil mist generation device was placed in the centerline and 20 m away from the return air grille horizontally, and its height was 1 m on the ground which was the same as the height of real cutting machine platform.

**Fig. 3:**
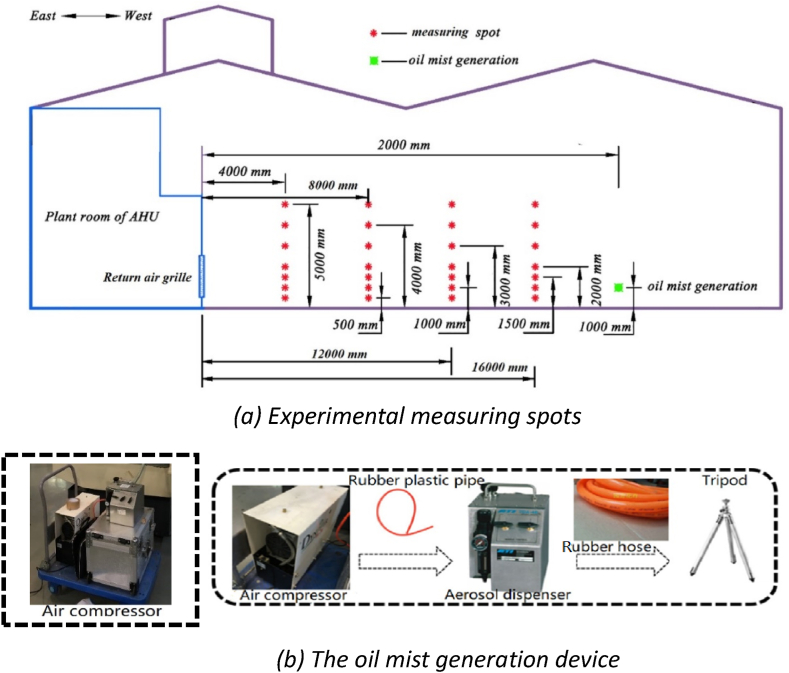
The oil mist generation device and experimental measuring spots in the full-scale workshop.

Fig. 3(a) demonstrates the experimental measuring spots in the full-scale workshop at vertical plane. The measuring spots were arranged on the vertical plane at 4 m, 8 m, 12 m, and 16 m away from the return air grille. Each plane had seven sensors in the midline vertically. Two sets of experiments were conducted under high-sidewall-nozzle (HSN) air supply mode and low-sidewall-vent (LSV) air supply mode. The air supply volume of the air conditioning system was calculated based on the cooling/heating load of the building and the required exhaust air volume. The corresponding airflow rate setting values were divided into three levels: high airflow rate (22500 m^3^/h), medium airflow rate (20000 m^3^/h), and low airflow rate (17500 m^3^/h). Under the HSN air supply mode, the eight air ejector nozzles at the height of 5.5 m were at working state while the other eight at the height of 7.5 m were not open. Details of the operational conditions are demonstrated in Table 3.

**Table 3:** The operational conditions of experiments.

Working condition	Ventilation mode	Air outlet		Airflow rate (m^3^/h)	Wind velocity (m/s)
		Area(m2)	Number		
HSN-17500	High-sidewall-nozzle (HSN) air supply mode	0.102	8	17500	5.97
HSN-20000				20000	6.82
HSN-22500				22500	7.68
LSV-17500	Low-sidewall-vent (LSV) air supply mode	1.650	8	17500	0.39
LSV-20000				20000	0.45
LSV-22500				22500	0.52

As shown in Fig. 3(b), the devices for oil mist generation included an air compressor, an ATI aerosol generator, a hard rubber plastic tube (8 mm in diameter), a rubber hose (20 mm in diameter), and a triangular bracket. The specifications of the air compressor and aerosol generator are shown in Table 4. The proportion of oil mist particles with different particle sizes is determined by the first-phase experiment in the case study machining workshop and is adjusted by the three knobs on the ATI aerosol generator. The particle generate rate was 0.02 kg/s and remained stable during the experiments.

**Table 4:** The technical specifications of the air compressor and aerosol generator.

Device	Manufacturer	Type	Range	Accuracy	Service conditions
Air compressor	DYNAIR	DA190/3C	0.1–100 mg/m^3^	±2%	+4∼+45 °C
Aerosol generator	America ATI	TDA-4B	0–10000 ppm	±3%	−20∼+50 °C

**Fig. 4:**
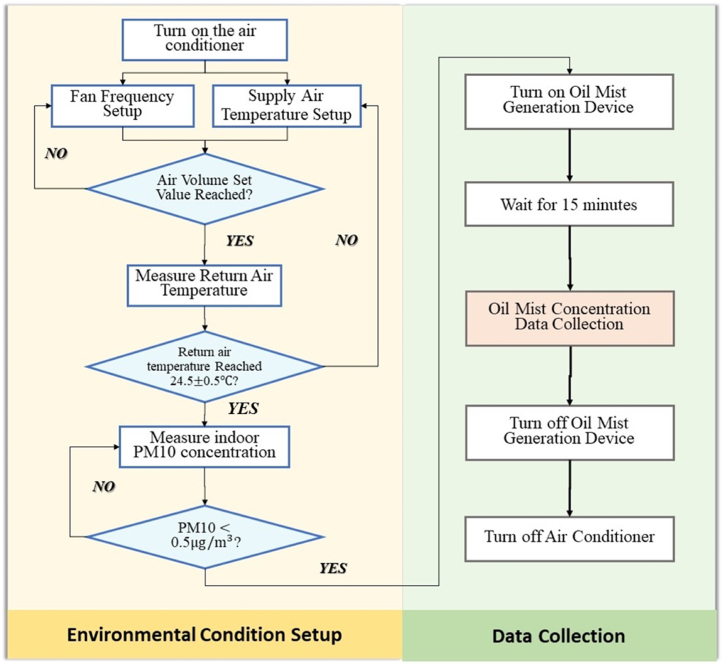
Flow chart of second-phase experiment.

The experimental procedure is diagrammatically presented in Fig. 4. Firstly, the air handling unit was turned on, and the fan frequency and supply air temperature were set up. Then, the actual value of the air supply volume was observed on the screen, and the fan frequency was adjusted until it reached the required air supply volume. Thirdly, the return air temperature was measured, and the supply air temperature was adjusted until the return air temperature value lay in the range of 24 ± 0.5 °C. After that, the concentration of PM10 particles was measured every 10 min until it was lower than 5 μg/m^3^. When the requirements for air supply volume, return air temperature, and PM10 concentration were all met, the oil mist generator was turned on. In order to reach the steady-state condition, the data collection of oil mist particle concentration started after the oil mist generator were turned on for 15 min, so that the oil mist particles were fully dispersed in space. After the data were collected, the oil mist generation device was turned off, and the test was ended. The air handling unit was also turned off after each test for 1 h to eliminate the interaction effects between two tests. In order to minimize the effect of background particles, the PM10 concentration level was continuously monitored, and the data collection would not start until the PM10 concentration value was stabilized below 5 μg/m^3^. The collected data included the oil mist concentrations and airflow velocities at each measuring spot, and return air temperature.

**Fig. 5:**
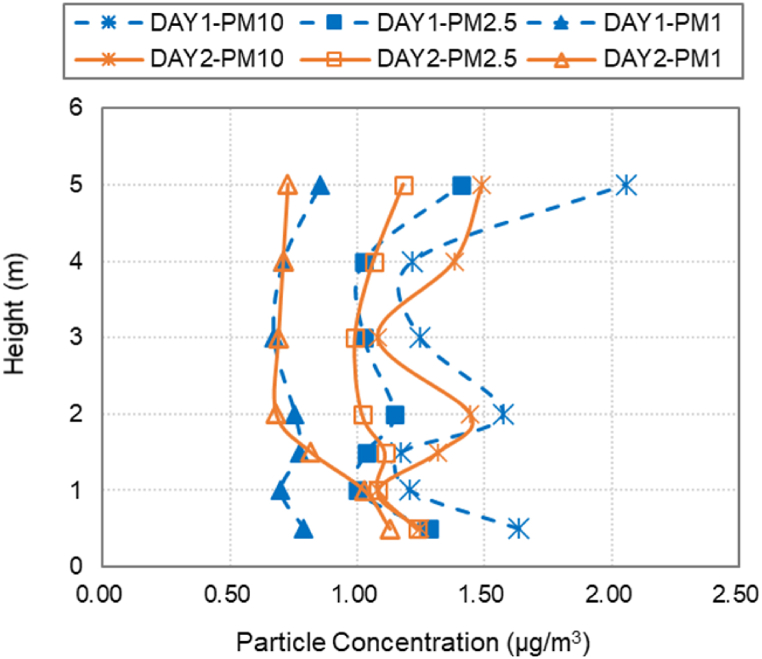
Comparison of particle concentration in the large space laboratory.

In order to eliminate the influence of the background concentration levels, a two-day preliminary test was conducted in the laboratory under the HSN air supply mode. On Day1 at 3:00 p.m., the vertical distribution of PM10, PM2.5, and PM1 concentrations at the same horizontal position were measured before the HVAC system and the oil mist generator were turned on for 3 h. On Day2 at 9:00 a.m. with the oil mist generator off, the concentration values of PM10, PM2.5 and PM1 at the same measuring spots were recorded. As shown in Fig. 5, the vertical distributions of each size of particular matter on the two days followed similar trends, while the background concentration values were almost the same and below 2.5 μg/m^3^. It indicates that due to the particle deposition effect, the oil mist particles on Day1 had little impact on the background particle concentration on Day2. Due to the comparably low background particle concentration levels, the influence of background particle concentration on the data collection under similar environmental conditions can be neglected.

## 3 Numerical methodology

### 3.1 Numerical method

The movement and diffusion of oil mist particles in airflow belong to multiphase flow issue, which can be solved by two numerical simulation methods: the Euler-Euler equations and the Euler-Lagrange equations. Euler–Euler (EE) models describe both the fluid and the particulate phase with transport equations on a globally fixed coordinate system-particles are not tracked in space and time; while Euler–Lagrange treat the fluid phase as a continuum while the dispersed phase is tracked in the Lagrange reference frame.

In this study, the ANSYS FLUENT software was used to simulate the oil mist particles diffusion in the machining workshop. The control equations consist of the continuity, momentum, and energy equations. The calculation uses Pressure-Based for steady-state calculation and solution, and uses the SIMPLE algorithm. The discretization scheme was set as a second order upwind for all variables. The near-wall surface is approximately solved through the standard wall equation. The convergence criterion for the simulation is that the residual is less than 10^−3^. The selected RNG k-ε model was used to describe the turbulent fluctuations of air flow. This study mainly focuses on the distribution and movement trajectory of oil mist particles, and the oil mist particles generated in the experiment have a volume fraction of less than 10% compared to air. Therefore, a discrete phase model based on the Euler-Lagrange model in FLUENT was selected. In the DPM model, the discrete random walking (DRW) model was used to account for the effect of the turbulent particle dispersion.

The control equations consist of the continuity, momentum, and energy equations. As shown in Eq. (1):

(1) $$\begin{array}{c} \frac{\partial p}{\partial t}+\frac{\partial \left(\rho v_{j}\right)}{\partial j}=S_{j} \end{array}$$

where ρ is the density of oil mist particles, j is 1, 2, 3, representing x, y, z three components respectively, t is the time of motion, S is quality original term, v is the velocity of the oil mist particles.

The equation of momentum for the oil mist particle in air can be obtained as Eq. (2):

(2) $$\begin{array}{c} \frac{\partial \left(\rho v_{j}\right))}{\partial t}+\frac{\partial \left(\rho v_{i}v_{j}\right)}{\partial x_{i}}=-\frac{\partial p}{\partial x_{i}}+\frac{{\partial \tau }_{ij}}{\partial x_{j}}+pg_{j}+F_{j} \end{array}$$

where $F_{j}$ is the component of the external body force in the j direction, $g_{j}$ is the component of gravity acceleration in j direction, p is static pressure, $pg_{j}$ is the mass-volume force.

$\tau_{ij}$ is the stress tensor at the fluid element, as shown in Eq. (3):

(3) $$\begin{array}{c} \tau_{ij}=\mu \left[\left(\frac{\partial v_{i}}{\partial x_{j}}+\frac{\partial v_{j}}{\partial x_{x}}\right)-\frac{2\partial v_{i}}{3\partial x_{i}}\delta_{ij}\right] \end{array}$$

(4) $$\begin{array}{c} \delta_{ij}=\left\{ \begin{array}{c} 1,i=j\\ 0,i\neq j \end{array}\right. \end{array}$$

The general form of the governing equations can be written as:

(5) $$\begin{array}{c} \frac{\partial \left(\rho \varphi \right)}{\partial t}+\mathrm{div}\left(pu\varphi \right)=\mathrm{div}\left(\Gamma grad\varphi \right)+S \end{array}$$

where φ represents the general variables, Γ the effective diffusion coefficient, and S is the source term.

### 3.2 Geometric model and boundary conditions

**Fig. 6:**
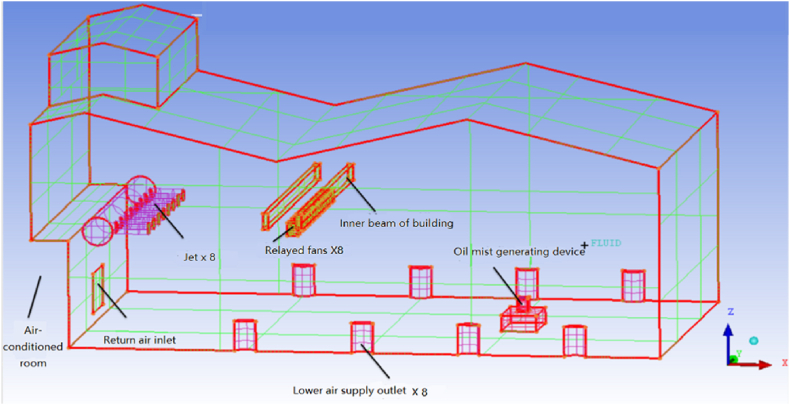
Machining workshop model.

The geometric model of the machining workshop is shown in Fig. 6. The unstructured grid was used for meshing, and the four different grid sizes of 2.01 million, 3.20 million, and 4.43 million were employed to conserve computing resources and their results verified for the grid independence analysis. The model with 3 million grid points was selected. The worst element had a skewness of 0.76. The enhanced wall treatment was applied to near wall regions, air ejector nozzles, and air supply vents in order to achieve fine grid and more accurate simulation results.

The set-ups of wind speed for the air nozzles, air supply vents were the same as the experimental study in Table 4. The corresponding wind speed for the return air grille in the simulation model are obtained from the experiments, which is 1.13 m/s, 1.29 m/s, 1.45 m/s respectively under the three working conditions of HSN and LSV air supply mode.

In this study, the surfaces of the oil mist generator were set as velocity inlets, and the escape boundary condition was adopted for the discrete phase. The natural air inlets of the windows were set as pressure inlet boundaries, and the mechanical air supply outlets as velocity inlets. The escape boundary condition was adopted for the discrete phase. Return air grille was set as velocity inlets, and the escape boundary conditions were adopted. All the wall surfaces, as well as the ground and ceiling, were set as adiabatic non-slip walls, and trap boundary conditions were adopted for the discrete phase. The measured data of oil mist particle concentration distribution in Table 1 working condition P1-1 (tool cutting metal aluminum block and adding lubricating oil lubrication) are analyzed, and the oil mist particle concentration under different particle sizes is obtained. The boundary conditions of the oil mist particle generation surface in the model are shown in Table 5.

**Table 5:** The boundary conditions of oil mist particle generating surface.

Oil mist (kg/s)	Particle size range	The proportion of oil mist (%)	The particle size of oil mist (μm)	Different sizes of oil mist (%)	The amount of oil mist (kg/s)
0.02	<1μm	40%	0.35	10%	0.002
			0.5	15%	0.003
			1.0	15%	0.003
	<2.5μm	40%	2.5	40%	0.008
	<10μm	20%	5.0	10%	0.002
			10	10%	0.002

## 4 Result and discussion

### 4.1 Experimental results and analysis

#### 4.1.1 Composition of oil mist in the case study

**Fig. 7:**
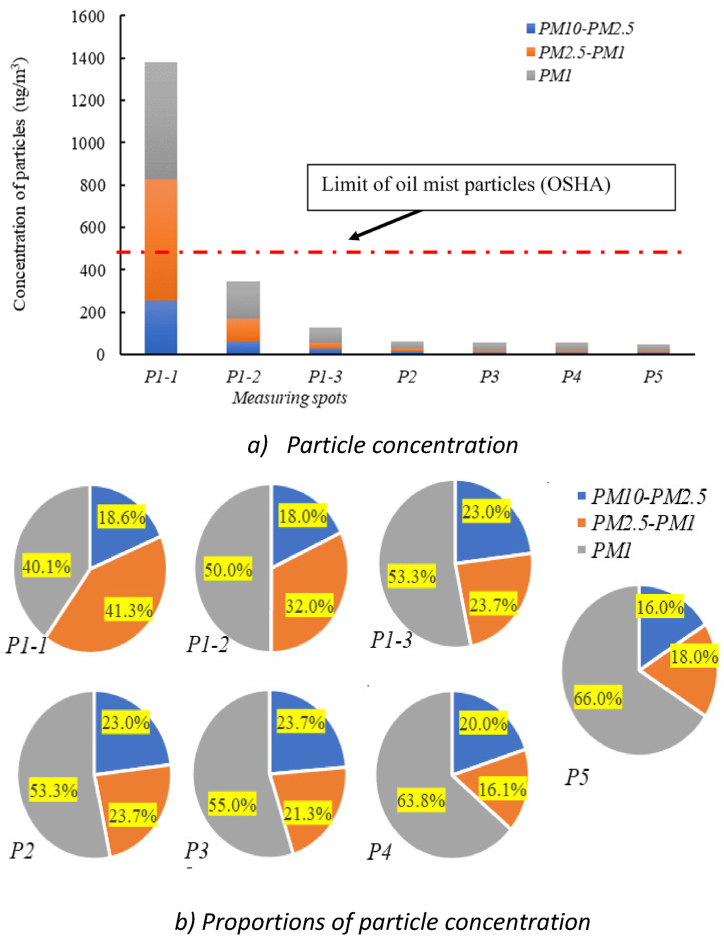
Composition of oil mist particles in machining workshop.

The composition of oil mist particles in machining workshop is presented in Fig. 7(a). During the cutting process, a large amount of oil mist was generated, and the average oil mist concentration of PM10, PM2.5 and PM1 at P1-1 was 1380.01 μg/m^3^, 1123.19 μg/m^3^ and 553.11 μg/m^3^, respectively. Although the concentrations of the oil mist particles for most measuring spots were below the OSHA standard limit for oil mist, the PM10 concentration at P1-1 during the cutting reached 1380.01 μg/m^3^, which is 2.76 times of the OSHA standard limit (0.5 mg/m^3^). At P1-2 after the cooling emulsion was added, the average oil mist concentration value of PM10, PM2.5 and PM1 was decreased to 344.44 μg/m^3^, 296.46 μg/m^3^, and 182.58 μg/m^3^, respectively. The fact that the particulate concentration test data of the other measuring spots did not exceed the OSHA standard limit is mainly due to that the processing of aluminum product is relatively simple and quick, and that few processing tasks in the remaining areas in the workshop.

Fig. 7(b) reveals that the concentration proportions of particles at different measuring spots were: 40.1–66.0% for PM1, 16.1–41.3% for PM2.5-PM1, and 16–23.7% for PM10-PM2.5 respectively. It is obvious that about 80% of the oil mist particles in the air of case study were with sizes less than 2.5 μm, which indicates that PM2.5 occupies the main component of oil mist particles during the mechanical processing.

Based on the above results, the oil mist generation devices in the second-phase full-scale experiments were set up, and the concentration ratios of PM10–2.5, PM2.5-1 and PM1 were 18.3 %, 38.4 % and 43.3 %, respectively, close to 18.6 % for PM10–2.5, 40.1 % for PM2.5-1, and 41.3 % for PM1, which can represent the actual oil mist compositions in real machining workshop. The errors between the concentration proportions of the case study and full-scale experiments were below 2%. To ensure that the proportions of particles in the six experiments were consistent, the three knobs on the oil mist generation devices were set steady after the first experiment started.

#### 4.1.2 Analysis of oil mist particles distribution in the full-scale experiment

**Fig. 8:**
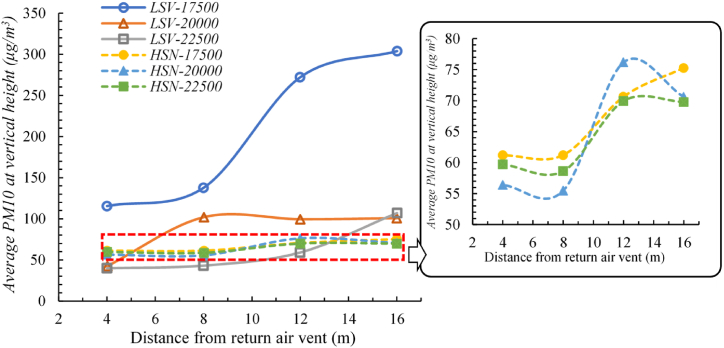
Average PM10 concentrations under different working conditions.

Fig. 8 depicts the general influence of different ventilation modes and air flow rate on the average concentration of PM10 of the oil mist. Under the high-sidewall-nozzle (HSN) air supply mode, the average PM10 concentrations at different positions were all at a low level of below 80 μg/m^3^ and showed little difference with each other. For HSN working conditions with different air flow rates of 17500 m^3^/h, 20000 m^3^/h, and 22500 m^3^/h, the ranges of PM10 concentration were 61.2–75.3 μg/m^3^, 55.5–76.2 μg/m^3^, 58.6–70.0 μg/m^3^ respectively. These facts prove that the HSN air supply mode is effective in removing PM10, and that increasing the air flow rate had little impact in reducing the concentration of PM10 under the high-sidewall-nozzle (HSN) air supply mode.

On the other hand, the average PM10 concentrations of different positions varied with each other under the low-sidewall-vent (LSV) air supply mode. The maximum PM10 concentration value of all positions reached to 303 μg/m^3^, while the minimum value was less than 50 μg/m^3^. For LSV working conditions with different air flow rates of 17500 m^3^/h, 20000 m^3^/h, and 22500 m^3^/h, the ranges of PM10 concentration is 115.4–303.8 μg/m^3^, 42.4–101.9 μg/m^3^, and 39.9–107 μg/m^3^ respectively. The above results indicate that the LSV air supply mode is less effective in removing PM10 compared with the HSN air supply mode, and the air flow rate has a large effect on reducing the PM10 concentration under this circumstance.

For detailed analysis, working conditions with air flow rate of 20000 m^3^/h were selected to discuss the influence of the ventilation modes on the distribution of oil mist particles.

**Fig. 9:**
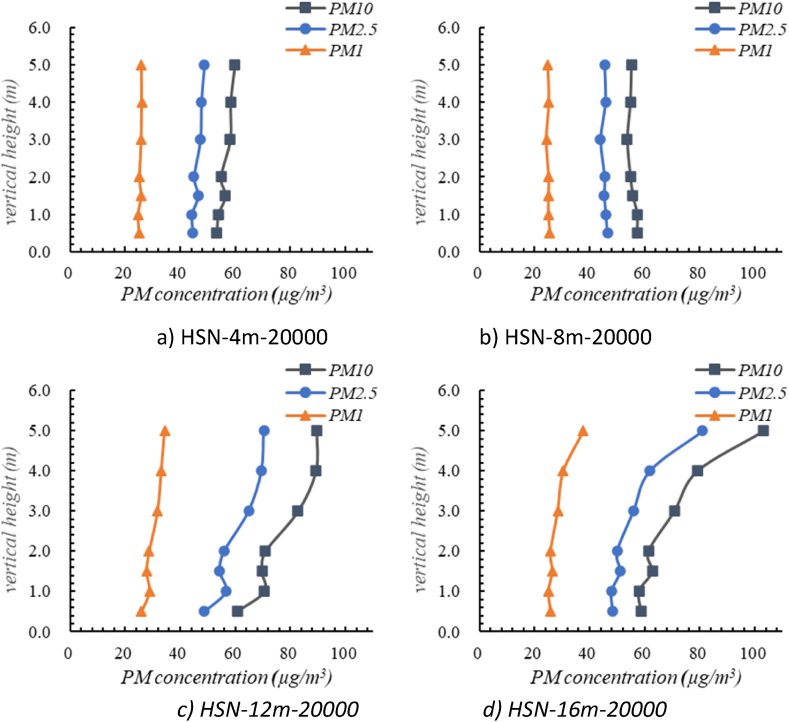
Distribution of oil mist particles under HSN air supply modes.

Fig. 9 shows the distributions of oil mist particles under the HSN air supply mode. It can be seen that the oil mist particles concentrations along the vertical directions at the same horizontal position followed the similar trends, which decreased slightly at first and then increased gradually. In general, the concentrations of oil mist particles at the height of 3–5 m were relatively higher than the concentrations at the height of 1–3 m, with the largest oil mist particles concentration less than 120 μg/m^3^. The maximum value of PM10 concentration was 103.09 μg/m^3^ (at position of 16 m away from the air return vent with the height of 5 m) and the minimum value is 53.34 μg/m^3^ (at position of 4 m away from the air return vent with the height of 1 m). In addition, the concentrations of oil mist particles with different heights at 4 m and 8 m measuring spots away from the air return vent showed little difference, while the concentration appeared obvious variance at measuring spots of 12 m and 16 m. Comparing the results along the horizontal direction, it can be found that the concentrations of oil mist particles near the air return vent were lower. The maximum values of PM10 at positions of 4 m, 8 m 12 m, 16 m away from the air return vent is 60 μg/m^3^, 57 μg/m^3^, 89 μg/m^3^, 103 μg/m^3^ respectively.

**Fig. 10:**
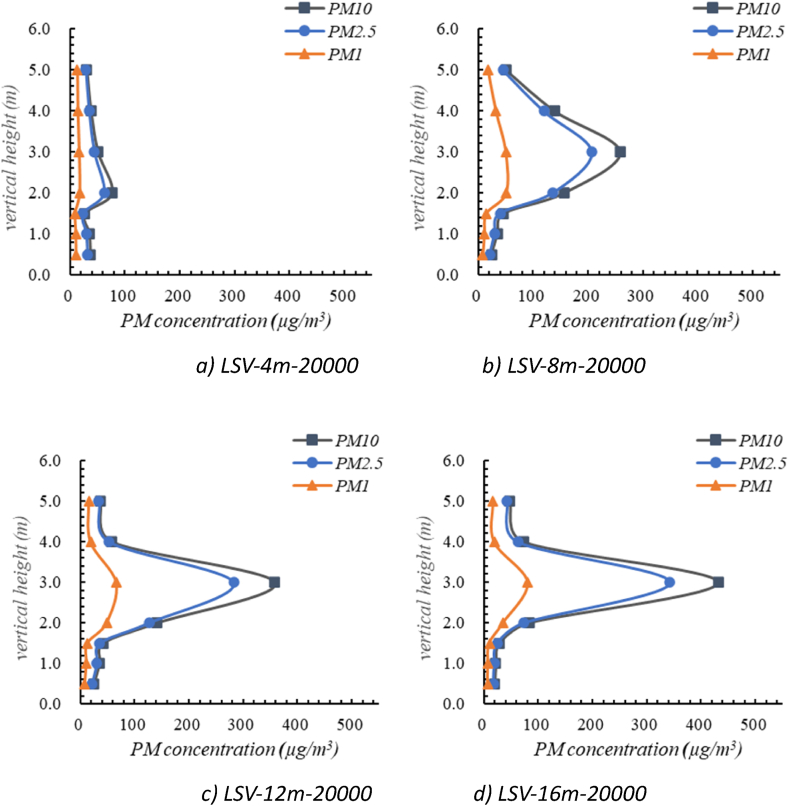
Distribution of oil mist particles under the LSV air supply mode.

As shown in Fig. 10, under the LSV air supply mode, the oil mist particles concentrations along the vertical directions at the same horizontal position also followed the similar trends, which increased at first and then decreased. In general, the concentrations of oil mist particles at the height of 3–5 m were relatively higher than those in the remaining area, with the peak value of oil mist particles concentration appears at height of 2∼3 m. The maximum concentration of PM10 was 432 μg/m^3^ (at the height of 3 m) and the minimum concentration was 20 μg/m^3^ (at the height of 0.5 m). The concentration ranges of PM10 at different positions (at position of 4 m, 8 m, 12 m, 16 m away from the air return vent) were 27–77 μg/m^3^, 24–259 μg/m^3^, 24–358 μg/m^3^, and 20–432 μg/m^3^, respectively. It can be concluded that the concentration ranges of oil mist particles along the vertical direction decreased as it approached the air return vent.

**Fig. 11:**
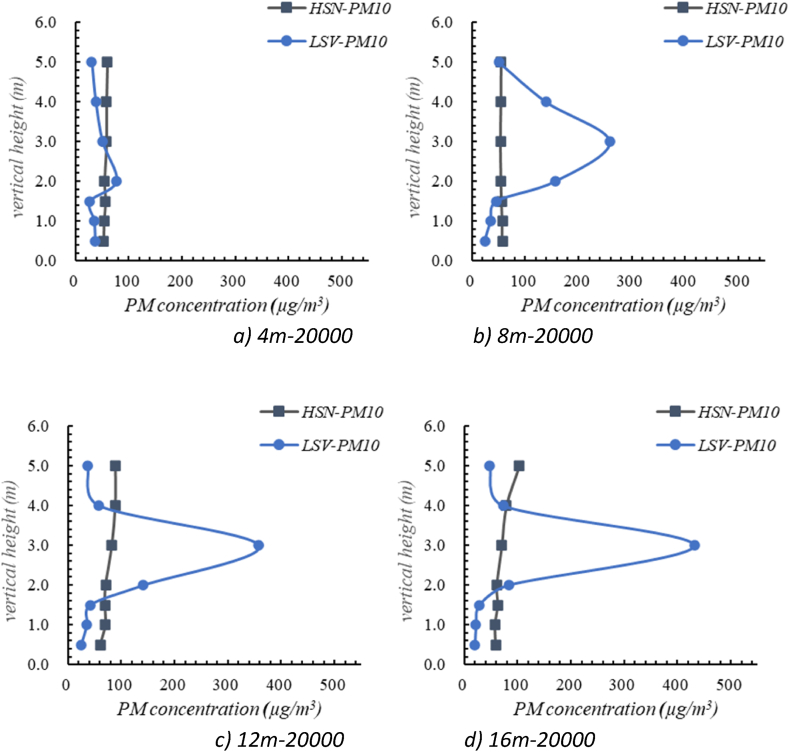
Distribution of PM10 under the different ventilation mode.

Fig. 11 illustrates the concentration distribution of PM 10 under different ventilation modes. The effectiveness of the two ventilation methods to remove oil mist particles is quantitatively analyzed by ventilation efficiency [38],

(6) $$\begin{array}{c} \eta =\frac{{C_{e}-C}_{s}}{C_{b}-C_{s}} \end{array}$$

Where η represents ventilation efficiency, $C_{e}$ is the average particle concentration at outlets, μg/m^3^; $C_{b}$ is the average particle concentration at respiratory height, μg/m^3^; $C_{s}$ is the average particle concentration at air supply inlet, μg/m^3^, which equals zero in this study.

Working conditions with air flow rate of 20000 m^3^/h were selected to discuss the influence of the ventilation modes on the distribution of oil mist particles (PM10). The results show that under the HSN-PM10-20000 mode, the ventilation efficiencies at different positions (at position of 4 m, 8 m, 12 m, 16 m away from the air return grille) were 96.3 %, 94.5 %, 74.3 % and 83.8 %, respectively. Under the LSV-PM10-20000 mode, the ventilation efficiencies were 92.5 %, 46.3 %, 52.8 % and 82.2 %, respectively.

In general, the PM10 concentration under LSV mode was higher than that under HSN mode at the vertical height of 2∼4 m, and lower than that under HSN mode at other levels. This is mainly due to that the wind direction and positions of air outlet have major impacts on the distribution of oil mist particles [39]. Under the LSV mode, the air supply vent was installed at lower position with the height of 1.5 m, and the return air grille was at a lower region (below 3 m). Therefore, the oil mist particles were mainly distributed at the middle region (2–4 m) in the workshop under the joint effect of gravity, buoyance and low-region air circulation. At lower-level regions near the air supply vent, the concentration of oil mist particles was less than 50 μg/m^3^ due to dilution of the constant fresh air. At regions higher than 4 m, the suspended oil mist particles gradually settled down into the middle region due to the gravity. While under the HSN mode, the air supply nozzles were located at the upper regions higher than 5.5 m, and the return air grille was same as LSV mode. The high velocity of supply air from the nozzles formed a good indoor air circulation which effectively reduced the concentration of oil mist particles and keep the concentration values at a stable level.

The above results address the effectiveness of the HSN air supply mode in removing oil mist particles, it was able to achieve high removal efficiency of oil mist particles. Under the LSV air supply mode the concentrations of oil mist particles in the workshop were distributed extremely uneven. The LSV air supply mode failed in removing most oil mist particles due to unfavorable air circulation and chaotic airflow under this ventilation mode. Hence, forming a good indoor air circulation is the key to control the distribution of oil mist particles, which is related to the location of the outlet.

### 4.2 Simulation results and analysis

#### 4.2.1 Simulation results under the HSN mode

**Fig. 12:**
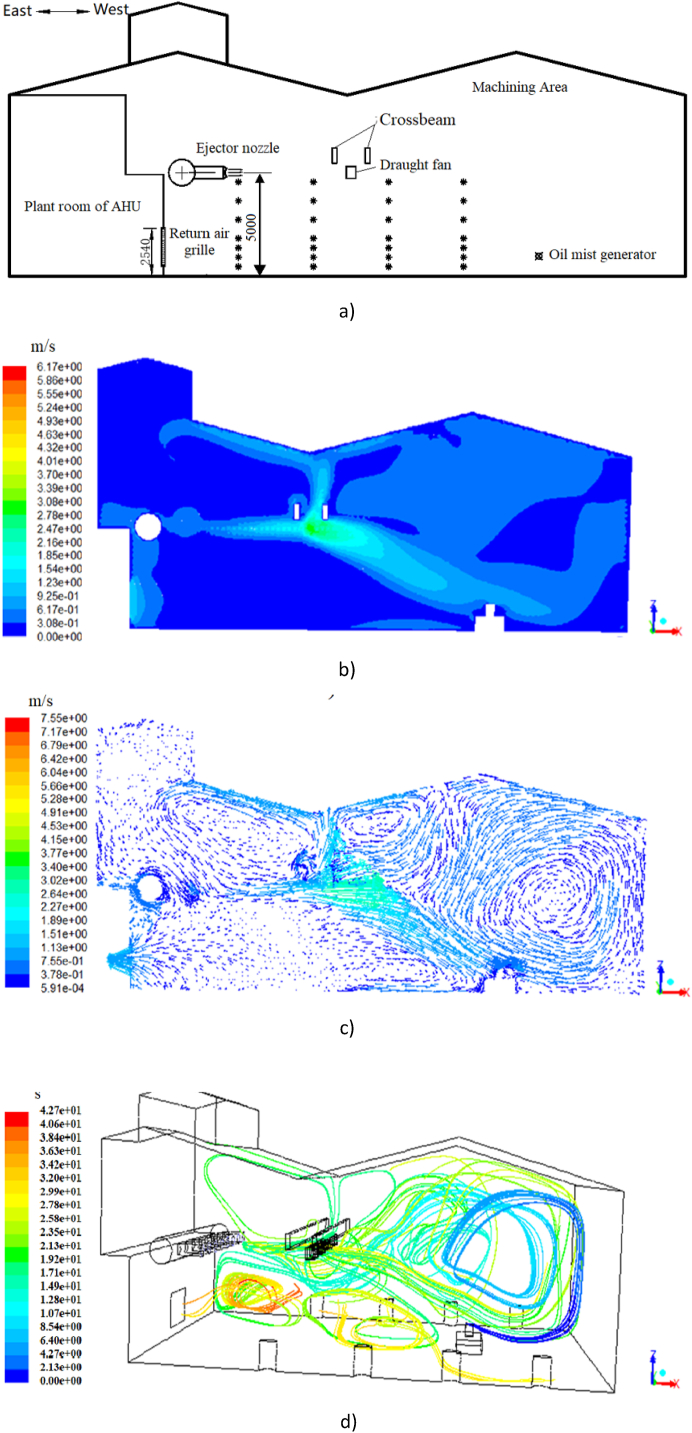
Results under the HSN mode (Plane y = 9) (a) Oil mist diffusion distribution experiment diagram. (b) Airflow velocity contour. (c)Airflow velocity vector. (d) Trajectory of particulate matter.

Fig. 12 presents the airflow velocity contour and vector of the section at y = 9 m (which is the plane where the 28 measuring points are located in the experiment) and the trajectory of particles under the high-sidewall-nozzle (HSN) mode (Fig. 12(a)) with an air volume of 17500 m^3^/h. As shown in Fig. 12 (b) and (c), the air ejected from the nozzle maintains a high wind speed at the height of 5.5 m which enables it to reach to the opposite area in the workshop. Due to the presence of beams and draught fans in the direction of air supply, the velocity and direction of the airflow changes which eventually leads the airflow move parabolically towards the ground. When the ejected air passing through the oil mist particle generator, the air velocity reduces to about 1 m/s. After that, a small portion of the airflow reverses its direction and moves to the return air grille or towards the ground; while a large portion of airflow forms a large eddy current before reaching to the West wall. In addition, the wind speed is almost unchanged in area lower than 5 m near the return air grille.

Fig. 12(d) depict the particle movement in the machining workshop under HSN mode. In general, the oil mist particles have similar distribution and trajectory with the airflow. When the oil mist particles move to the position of the crossbeam, a small part of the oil mist drifts along the gap of the beam to the upper space, indicating that if the indoor air distribution is not uniform enough, the oil mist particles can easily spread from the space not covered by the airflow to the external area. The trajectory of particles in HSN mode is basically in the range of large airflow velocity (3–5 m vertical height range), which is located in the upper part of the working area. The farther away from the return air grille, the more obvious this trend is. From the perspective of force analysis, the drag force is the dominant force affecting the motion of oil mist particles, which is greater than the influence of gravity and Brownian force on particles.

**Fig. 13:**
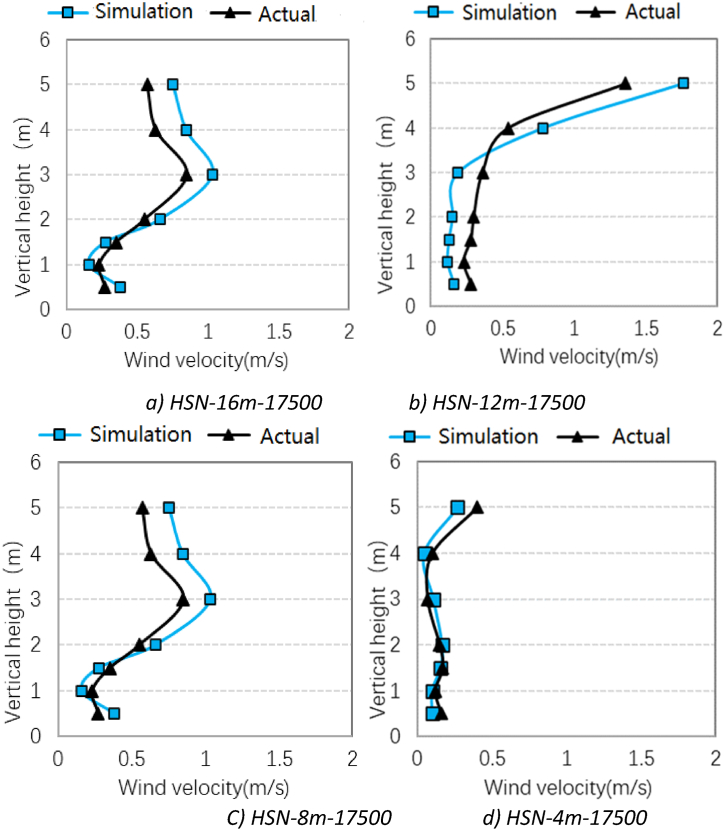
Comparison of experimental and simulated data-HSN mode.

In order to validate the simulation model, the simulated wind speed values of 28 measuring points were compared with the obtained experimental data. Fig. 13 shows the comparison of the simulated and measured wind speed values at the positions of 16 m, 12 m, 8 m, and 4 m from the return air grille under the air supply volume of 17500 m^3^/h. It can be seen that the wind speed values of the measured data and simulated results at each point in space are almost equal, and the trend of vertical height is also consistent with each other, which prove the reliability of the simulation results.

#### 4.2.2 The simulation results under the LSV mode

**Fig. 14:**
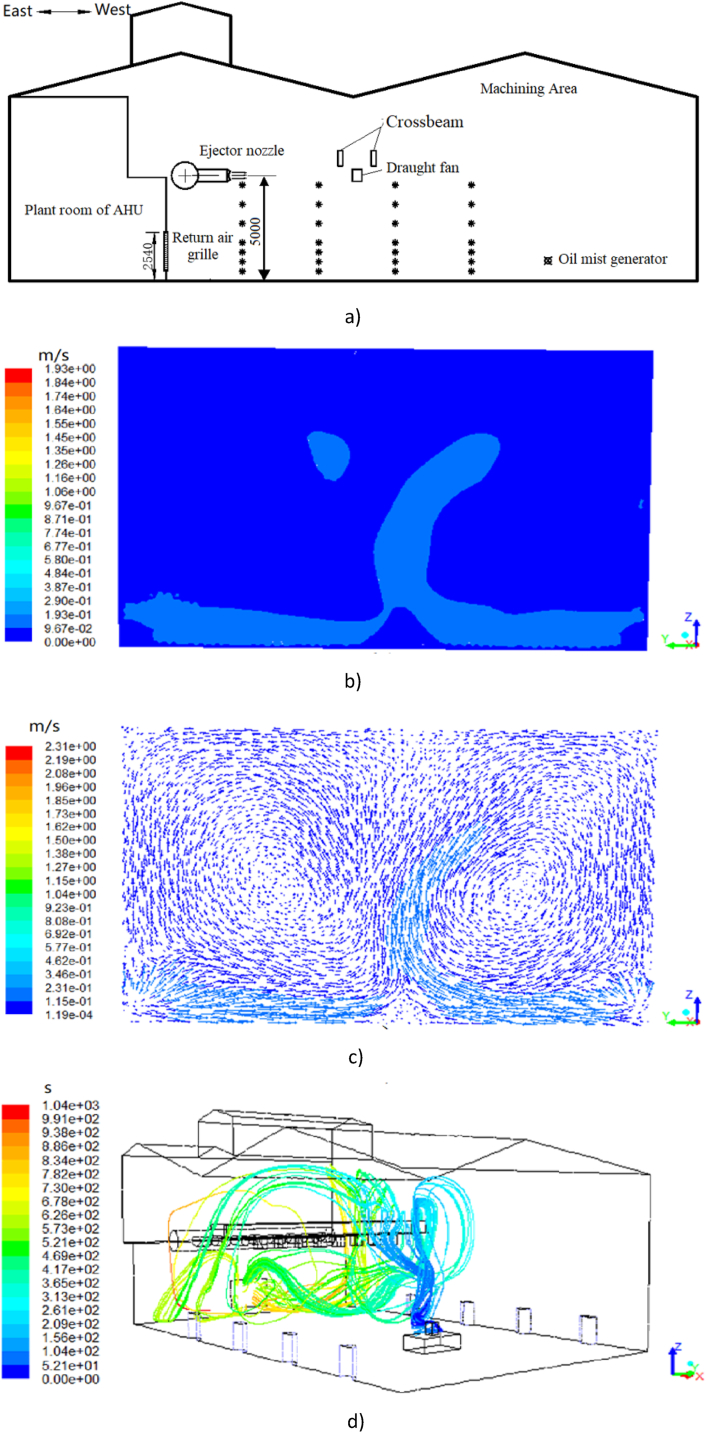
Results under the LSV mode *(a) Oil mist diffusion distribution experiment. (b) Contour of airflow velocity (Plane y = 9). (c) Vectors of airflow velocity (Plane x=24.2). (d) Trajectory of particulate matter*.

Fig. 14(b) demonstrates the simulation results under the LSV mode. In general, the airflow velocity is lower than 0.1 m/s when the height is lower than 1 m or higher than 4 m above the ground, and the higher airflow velocity mainly occurs at the height of 1∼4 m above the ground with the highest near the return air grille. As shown in Fig. 14 (c), two symmetrical vortex air flows are formed at x = 24.2 m (which is the plane where the oil mist generator is located in the experiment) in the building under the LSV mode. After the air comes out at a low speed from the eight air supply vents on the both sides of the walls, the airflow moves towards the middle of the building. After the two streams of airflow meet in the middle, the airflow velocity decreases and the airflow moves upward with a velocity of about 0.1 m/s. Afterwards, the airflow is divided into two streams at a vertical height of 3.5 m and moves from the middle to the sides at a low speed respectively.

Fig. 14(d) shows the trajectory of oil mist particles. The oil mist particles first move upward, spread to both sides at a vertical height of 3.5 m, and then gradually fall down. Most of the particles move randomly at the height ranging from 1 m to 4 m. The trajectory of particles in LSV mode is basically in the same range of airflow movement (1–4 m height) which is also the working area of the workshop. The oil mist particles are mainly distributed in the range of the working area under the LSV mode, which is not recommended in terms of the occupants’ health. This is due to the low airflow velocity, the influence of gravity and Brownian force on the motion of particles is larger than the drag force. Under the LSV mode, higher airflow velocity should be considered to achieve better pollutant control effect.

**Fig. 15:**
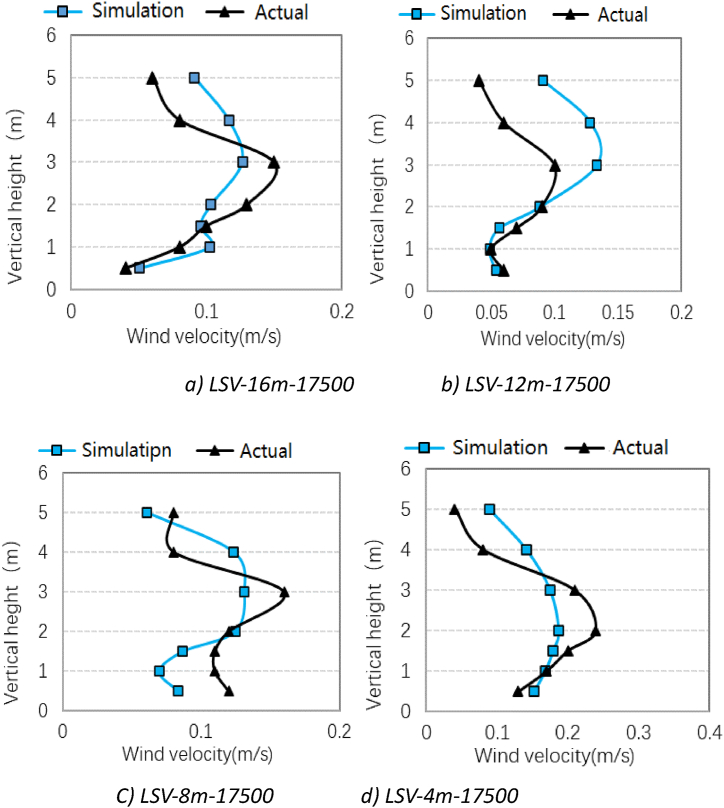
Comparison of experimental and simulated data-LSV mode.

Fig. 15 shows the comparison of the simulated results and the measured data at the positions of 16 m, 12 m, 8 m and 4 m from the return air grille when the air supply volume is 17500 m^3^/h. It can be seen that the measured data and simulation results of wind speed at each measuring point in space are slightly different under the LSV mode, but the trend of vertical height is basically the same, indicating that the simulation results are reliable. It can also be found that the airflow velocity in the working area is less than 0.4 m/s, which can meet the criteria of thermal comfort in Chinese national standards in terms of airflow velocity.

## 5 Conclusion

In this study, both data collections in a real processing workshop and full-scale experiments in a large-space laboratory were carried out. The distributions of oil mist particles concentrations under two ventilation modes were investigated. The main conclusions are drawn as follow.

1) PM2.5 occupied the main component of oil mist particles in the machining processing of the real process workshop in the case study.
2) The high-sidewall-nozzle air supply mode was able to provide high removal efficiency of oil mist particles, with the largest particle concentration lower than 150 μg/m^3^ which occurred at the height of 5 m. Under the HSN air supply mode, increasing air flow rate did not result in obvious effect in reducing the oil mist concentration.
3) Compared with the high-side-nozzle air supply mode, the low-sidewall-vent air supply mode in the experiments resulted in suspended oil mist particles and uneven distribution of oil mist concentrations, with the peak concentration value reached 432 μg/m^3^ which occurred in the middle area at the height of 2∼3 m. Increasing air flow rate can be very effective in reducing the oil mist concentration under the LSV air supply mode.
4) Regardless of ventilation modes, the concentrations of oil mist particles along the horizontal direction decreases as it approached the return air grille.
5) Under the HSN air supply mode, the oil mist particles show good following characteristics to the airflow; while under the LSV air supply mode, the oil mist particles may not show good following characteristics when the airflow velocity is low.

This study focuses on the influence of two typical ventilation modes with different air supply volumes on oil mist particles diffusion in machining workshop. However, there are some limitations with the current study which could be investigated in future. Different influencing factors on the effectiveness of air pollutant removal could be further investigated, such as temperature gradient and high humidity of the indoor environment, the positions of the air inlets and outlets (i.e., the asymmetry of the air suppliers). In addition, the impacts of the two ventilation modes on the distributions of particle concentrations in the occupants' breathing zone should be studied. Although the simulated trajectories of the particles were provided as supplementary results, future research is encouraged to be carried on the experimental validation of the particle pathways by using particle tracking velocimetry method.

## Data availability statement

Data will be made available on request.

## CRediT authorship contribution statement

**Yilin Li:** Writing – original draft, Visualization, Funding acquisition, Conceptualization. **Shiji Zong:** Writing – review & editing, Investigation, Formal analysis. **Huaiwang Jing:** Writing – review & editing, Formal analysis. **Nan Gao:** Methodology, Data curation. **Heping Ye:** Software. **Jianbo Chen:** Supervision, Funding acquisition.

## Declaration of competing interest

The authors declare that they have no known competing financial interests or personal relationships that could have appeared to influence the work reported in this paper.
